# The correlation between the MRI-evaluated ectopic fat accumulation and the incidence of diabetes mellitus and hypertension depends on body mass index and waist circumference ratio

**DOI:** 10.1371/journal.pone.0226889

**Published:** 2020-01-27

**Authors:** Joanna Pieńkowska, Beata Brzeska, Mariusz Kaszubowski, Oliwia Kozak, Anna Jankowska, Edyta Szurowska

**Affiliations:** 1 II Department of Radiology – Faculty of Health Sciences, Medical University of Gdansk, Gdansk, Poland; 2 Department of Biology and Pharmaceutical Botany, Faculty of Pharmacy, Medical University of Gdansk, Gdansk, Poland; 3 Department of Human Physiology, Faculty of Health Sciences, Medical University of Gdansk, Gdansk, Poland; 4 Institute of Statistics, Department of Economic Sciences, Faculty of Management and Economics, Gdansk University of Technology, Gdansk, Poland; 5 I Department of Radiology – Faculty of Medicine, Medical University of Gdansk, Gdansk, Poland; 6 Department of Radiology, University Clinical Centre in Gdansk, Gdansk, Poland; University of Central Florida (UCF), UNITED STATES

## Abstract

The widespread presence of overweight and obesity increases with every decade, and the number of people with body mass index (BMI) >30 kg/m^2^ has doubled in the last 30 years. The aim of the study is to assess the correlation between MRI-evaluated ectopic fat accumulation in pancreas, skeletal muscles and liver and the incidence of type 2 diabetes and hypertension, depending on BMI and waist circumference ratio. This prospective study included 267 consecutive patients who were referred to abdominal MRI and underwent a standard clinical assessment with BMI and waist circumference ratio calculation. Ectopic fat accumulation in pancreas, skeletal muscles and liver was evaluated in magnetic resonance imaging using the fat-water separated Dixon imaging. There were statistically significant differences in mean steatosis of all assessed organs in the group of patients with type 2 diabetes or hypertension in comparison to the non-diabetic group as well as to the group without hypertension. It has been observed that pancreas and skeletal muscles are more susceptible to fat accumulation than liver. According to our results, there is a relation between the fat content in muscles, pancreas and liver, the incidence of type 2 diabetes and hypertension and also body mass index and waist circumference ratio. We believe that future studies should aim to determine whether the use of fat content measurement in certain organs could be used as a biomarker that can enable early detection of reversible metabolic changes, as well as their subsequent monitoring.

## Introduction

The prevalence of overweight and obesity increases with every decade, and the number of people with body mass index (BMI) >30 kg/m^2^ has doubled in the last 30 years [1, 2]. It has been proven that the presence of obesity is associated with life-threatening diseases, such as metabolic syndrome (MetS), cardiovascular disease and cancer, which makes obesity one of the most important health problems of the 21st century [2–6]. MetS, which consists of at least three of the five abnormalities, that include diabetes or elevated fasting glucose level, central obesity, high triglyceride level (TG), decreased high-density lipoprotein cholesterol level (HDL) and high blood pressure, is a cluster of the most dangerous risk factors for myocardial infarction [5, 7–10]. According to the World Health Organization (WHO) data, MetS can currently be found in approximately 20–25% of the adult population worldwide, and it is estimated that this group of people is three times more likely to develop a myocardial infarction or stroke compared to the rest of the population. Additionally, people with MetS present a 5-fold higher risk of developing type 2 diabetes (T2DM) [7]. The incidence of diabetes is expected to double by 2025, and in 2030 diabetes will be the seventh cause of death in the world.

Until now, T2DM has been regarded as an inexorable, inevitably progressive process. However, it is currently understood as a potentially reversible metabolic state caused by chronic, excessive, ectopic fat accumulation [3, 11]. Recent studies evaluating the effect of weight loss on fat content in the pancreas have shown that there is a reduction in ectopic fat infiltration, along with a reduction in body mass. It is accompanied by a reversal of T2DM, improvement in insulin sensitivity and normalization of glucose metabolism [2, 5, 12, 13]. These data indicate that lifestyle modification can reduce morbidity and mortality due to MetS-related diseases, including type 2 diabetes.

Insulin resistance is a good predictor for the clinical onset of T2DM and precedes occurring of the disease for many years.

In the case of obesity, the level of circulating triglycerides and free fatty acids (FFAs) at some point begins to exceed the metabolic capacity of adipose tissue, leading to their excessive accumulation in other organs, including liver, pancreas and skeletal muscles. That results in serious metabolic and clinical consequences as increased lipid accumulation inhibits insulin-mediated glucose uptake and thereby reduces insulin sensitivity of the organs [1–3, 5, 12, 14–17].

It seems that magnetic resonance imaging MRI examination which allows non-invasive assessment of lipid infiltration in various organs may become an ideal tool that can help in the struggle against the effects of obesity. We hope that by showing a correlation between fat content in the pancreas, liver and skeletal muscles and the occurrence of metabolic disorders developing in the course of obesity, MRI will allow early detection of reversible metabolic changes as well as their further monitoring. We believe that in the future, the method could also be used as a biomarker that indicating the development of pre-diabetes insulin resistance.

The aim of the study is to assess the correlation between MRI-evaluated ectopic fat accumulation in pancreas, liver and skeletal muscles and the incidence of type 2 diabetes and hypertension, depending on body mass index and waist circumference ratio.

## Materials and methods

The study protocol was approved by the Local Ethical Committee (Independent Bioethics Commission for Research of the Medical University of Gdansk, Poland). All participants gave their written consent to participate in the study after they have been informed about the methods and purposes of the examination. All authors had been given access to the study data and then reviewed and approved the final manuscript.

This prospective study includes 267 consecutive patients of Caucasian origin (119 men and 148 women) who were referred to abdominal MRI for various non-malignant reasons (as non-specific abdominal pain, pancreas or liver observation) and underwent a standard clinical evaluation, a physical examination and biochemical tests. All examinations were performed between January and August 2018. We measured the height and body weight of the participants to calculate the Body Mass Index by dividing the weight in kilograms by the square of the height in metres:

$$BMI=\frac{weight\;\left(kg\right)}{\left[height\;\left(m\right)2\right]}$$

The commonly accepted BMI ranges were used: underweight—less than 18.5 kg/m^2^, normal weight from 18.5 to 24.9, overweight from 25 to 29.9 and obese ≥ 30 kg/m^2^. Waist circumference was measured at a level midway between the lowest rib margin and the iliac crest of the pelvis in the horizontal position.

MetS was defined according to the criteria modified by the National Cholesterol Education Program Adult Treatment Panel III Guidelines [18].

Central obesity was defined using the ethnicity-specific values for waist circumference in Europe which is ≥ 80 cm for women and ≥ 94 cm for men.

Because of the different reference values of the waist circumference depending on the gender, a universal for both groups numerical value defined in per cent and called Waist Circumference Ratio (WCR) was introduced [19]. WCR [%] represents the ratio of the waist circumference of the patient to the reference value, which is 80 cm for a woman and 94 cm for a man. In other words:

$$WCR=\{\begin{array}{ll} \frac{waist\;circumference}{80}\cdot 100\% & for\;woman\\ \frac{waist\;circumference}{94}\cdot 100\% & for\;man \end{array}$$

For both women and men, the WCR for limit values of waist circumference is 100%, with normal waist circumference <100%, for central obesity >100%.

To determine the deviation from the normal values of the waist circumference in centimeters, we used a variable which was labelled as circumference deviation (CD). The CD represents the deviation of the waist circumference from the reference value and is the difference expressed in centimeters between the patient's waist circumference and the reference value (different for women and men—80 vs 94 cm).

Elevated TG level was confirmed when the range limit of 150 mg/dL was exceeded. As a low HDL cholesterol level less than 40 mg/dL for men and less than 50 mg/dL for women was assumed. Hypertension (HT) was diagnosed if systolic or diastolic blood pressure was elevated above 130/85 mmHg or in the case of previously diagnosed HT treatment. Impaired fasting glucose level was defined as ≥ 100 mg/dL or in the case of previously diagnosed type 2 diabetes mellitus treatment.

Abdominal MRI examinations were performed using 1.5 T Siemens Magnetom Aera system. All subjects underwent an identical imaging protocol on the same MR scanner.

Ectopic fat accumulations in the pancreas, liver and skeletal muscles were evaluated in MRI using the fat-water separated Dixon imaging technique which leads to fat and water signal selection using the chemical shift between the resonance frequencies of protons bound in the fat and water. This technique automatically generates pixel-wise parametric maps that specify fat content based on 'only water' and 'only fat' images. After selecting a specific area in the organ, using regions of interest (ROIs), the numerical values of the signal intensity (SI) on the generated fat and water images can be obtained. Based on the acquired signal intensity values, the content of fat fractions in individual organs can be calculated.

Quantitative assessment of the fat accumulation was achieved by computing the percentage value of the fat fraction which is the fat signal intensity divided by the sum of the fat and water signals and then multiplied by 100. In other words fat fraction (FF) is computed as follows:

$$FF=\frac{SI\left(F\right)}{SI\left(W+F\right)}\;x\;100\%$$

where SI is the signal intensity contributions from water (W) and fat (F). This method correlates better with histopathologically confirmed steatosis than conventional dual in-phase and out-of-phase magnetic resonance imaging.

Regions of interest (ROIs) were placed manually within the target organs and then copied to ensure that the size and location are the same on both fat-only (F) and water-only (W) images.

To measure pancreatic fat and water signals, 3 circular regions of interest (ROIs) were drawn into three anatomical parts of the pancreas. To avoid contamination from volume averaging with extrapancreatic adipose tissue ROIs were placed in the pancreatic parenchyma in a way that they would be surrounded by pancreatic tissue not only within the imaging plane but also on the slices above and below. When ROIs were selected the pancreatic duct and vessels were also carefully avoided. The mean of all ROIs in each part of the pancreas (the head, body and tail) was calculated to determine the average fat fraction.

The hepatic fat content was assessed by using ROIs which were as large as possible with a homogeneous parenchyma signal, in order to avoid inclusion of visible extra- and intrahepatic vessels and enlarged bile ducts.

**Fig 1:**
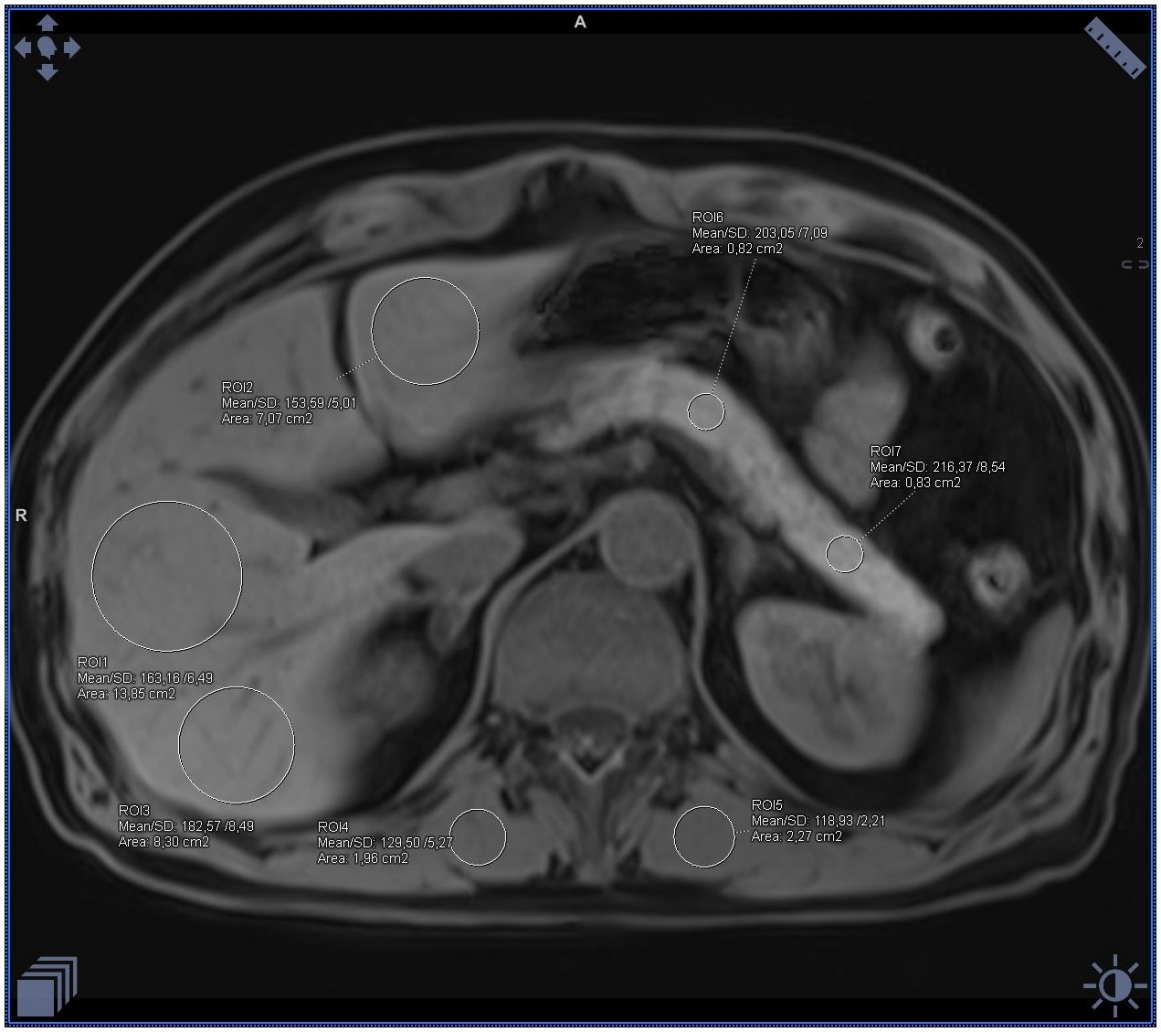
Example of the ectopic fat accumulation measurements in the pancreas, liver and skeletal muscles on the fat-water separated Dixon image in a healthy patient with BMI 21 and normal waist circumference. Measurements visible on the image with fat saturation represent only water signal (calculated mean fat fraction: liver– 2%, pancreas– 4%, muscles– 3%).

**Fig 2:**
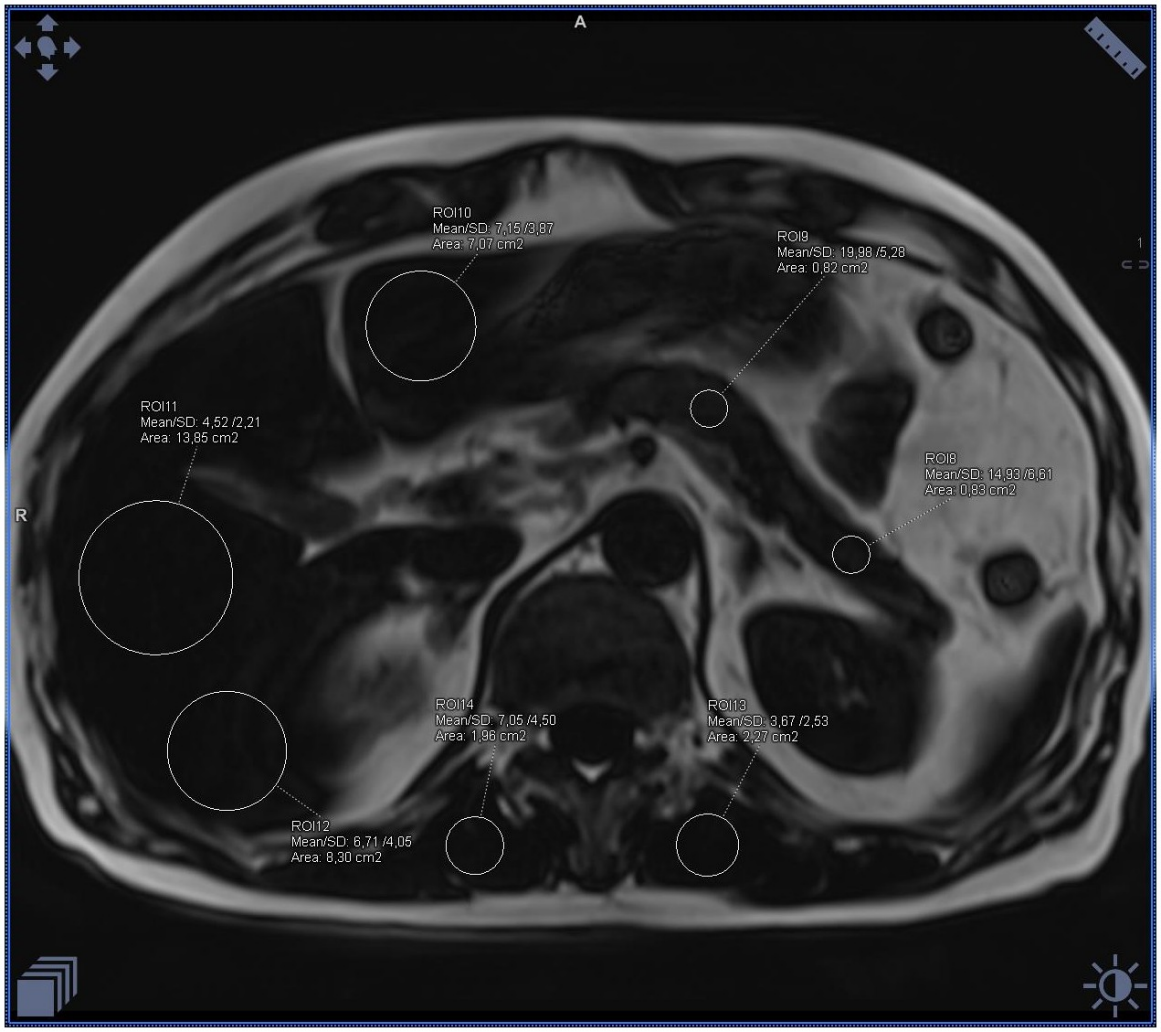
Example of the ectopic fat accumulation measurements in the pancreas, liver and skeletal muscles on the fat-water separated Dixon image in a healthy patient with BMI 21 and normal waist circumference. Measurements visible on the image without fat saturation represent the sum of the fat and water signal (calculated mean fat fraction: liver– 2%, pancreas– 4%, muscles– 3%).

**Fig 3:**
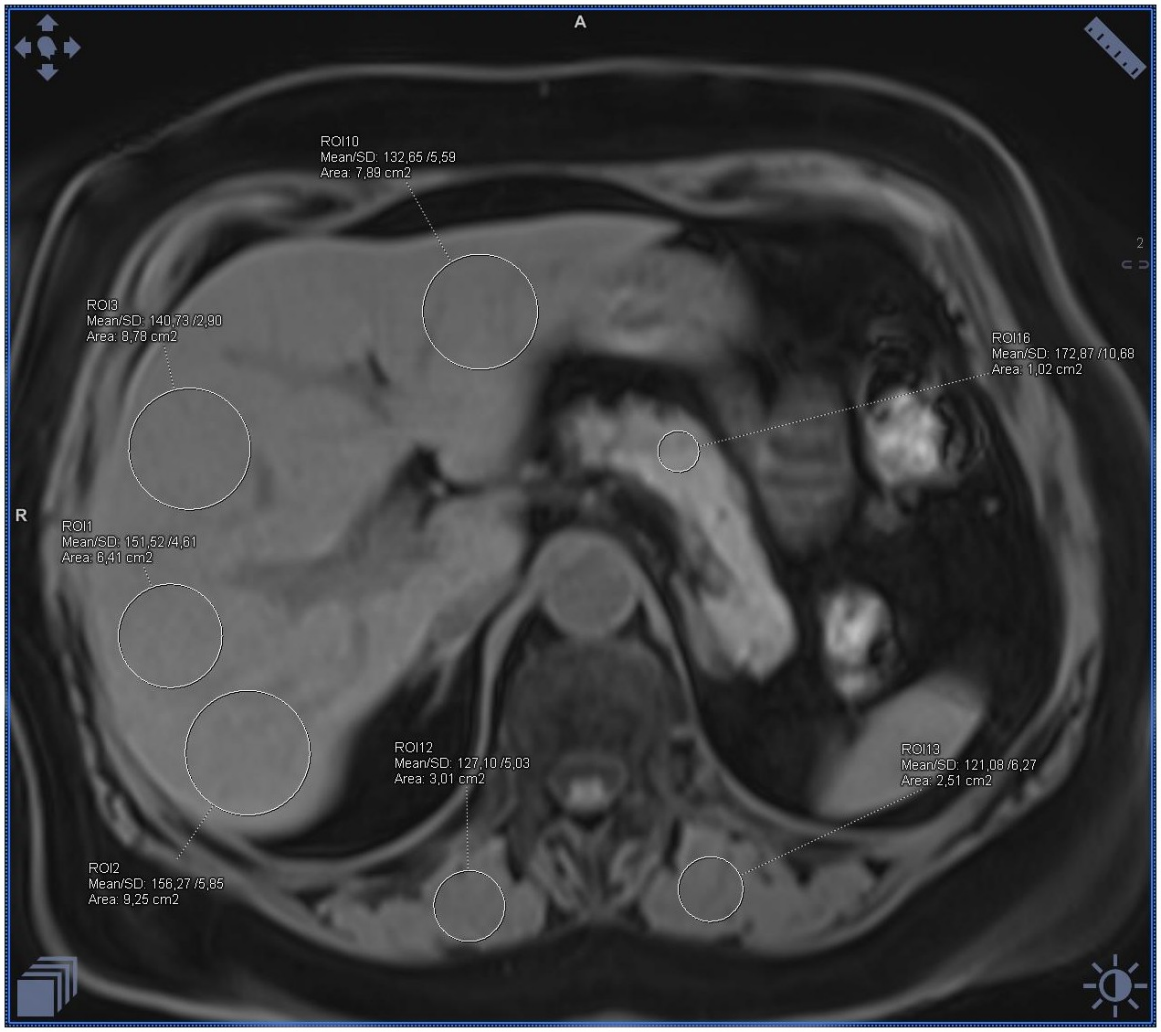
Example of the fat accumulation measurements in the liver and skeletal muscles on the fat-water separated Dixon image in a patient with BMI 29 and increased waist circumference, in whom diabetes and hypertension were diagnosed. Measurements visible on the image with fat saturation represent only water signal (calculated mean fat fraction: liver– 21%, pancreas– 13%, muscles– 11%).

**Fig 4:**
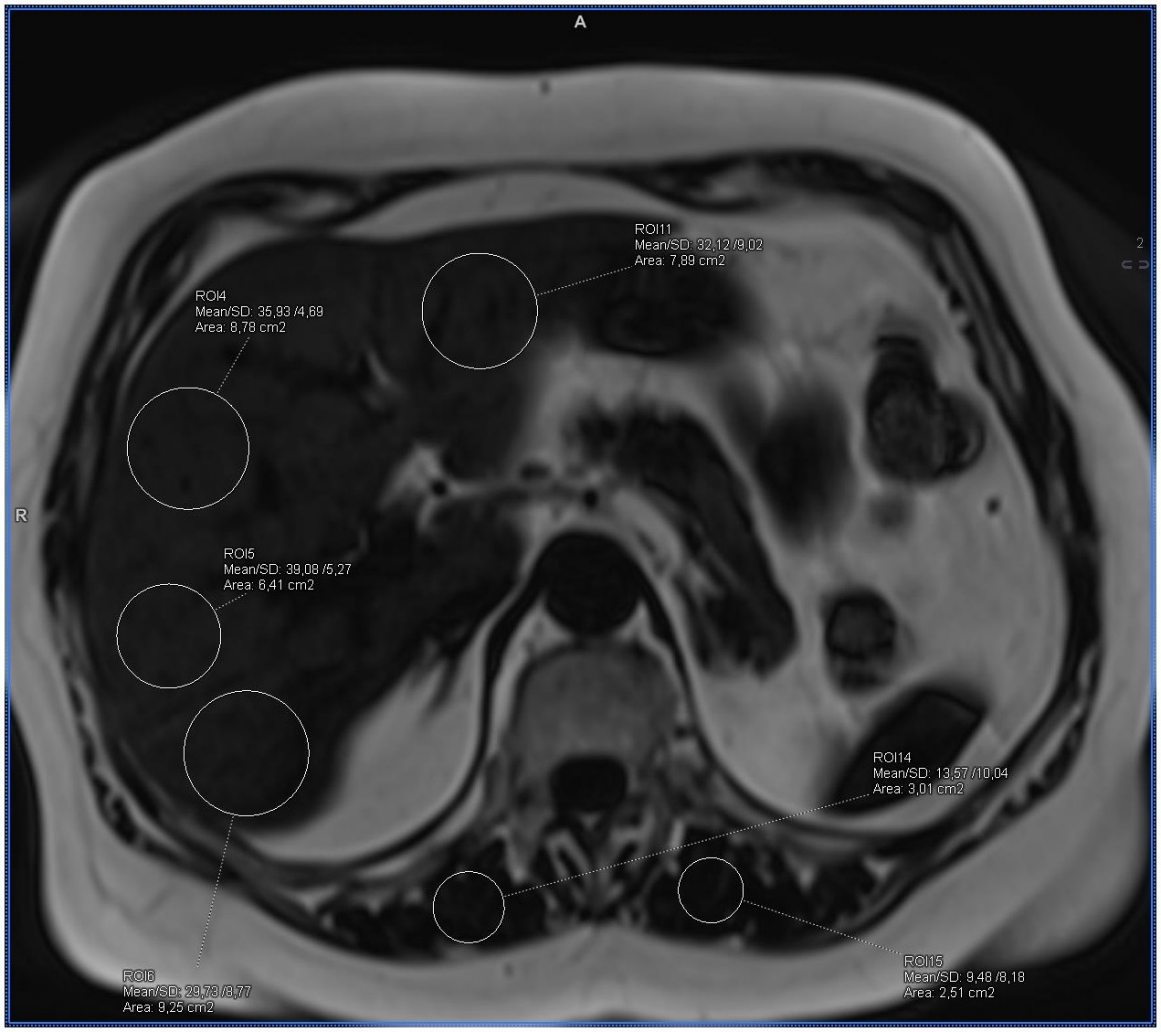
Example of the ectopic fat accumulation measurements in the liver and skeletal muscles on the fat-water separated Dixon image in a patient with BMI 29 and increased waist circumference, in whom diabetes and hypertension were diagnosed. Measurements visible on the image without fat saturation represent the sum of the fat and water signal (calculated mean fat fraction: liver– 21%, pancreas– 13%, muscles– 11%).

**Fig 5:**
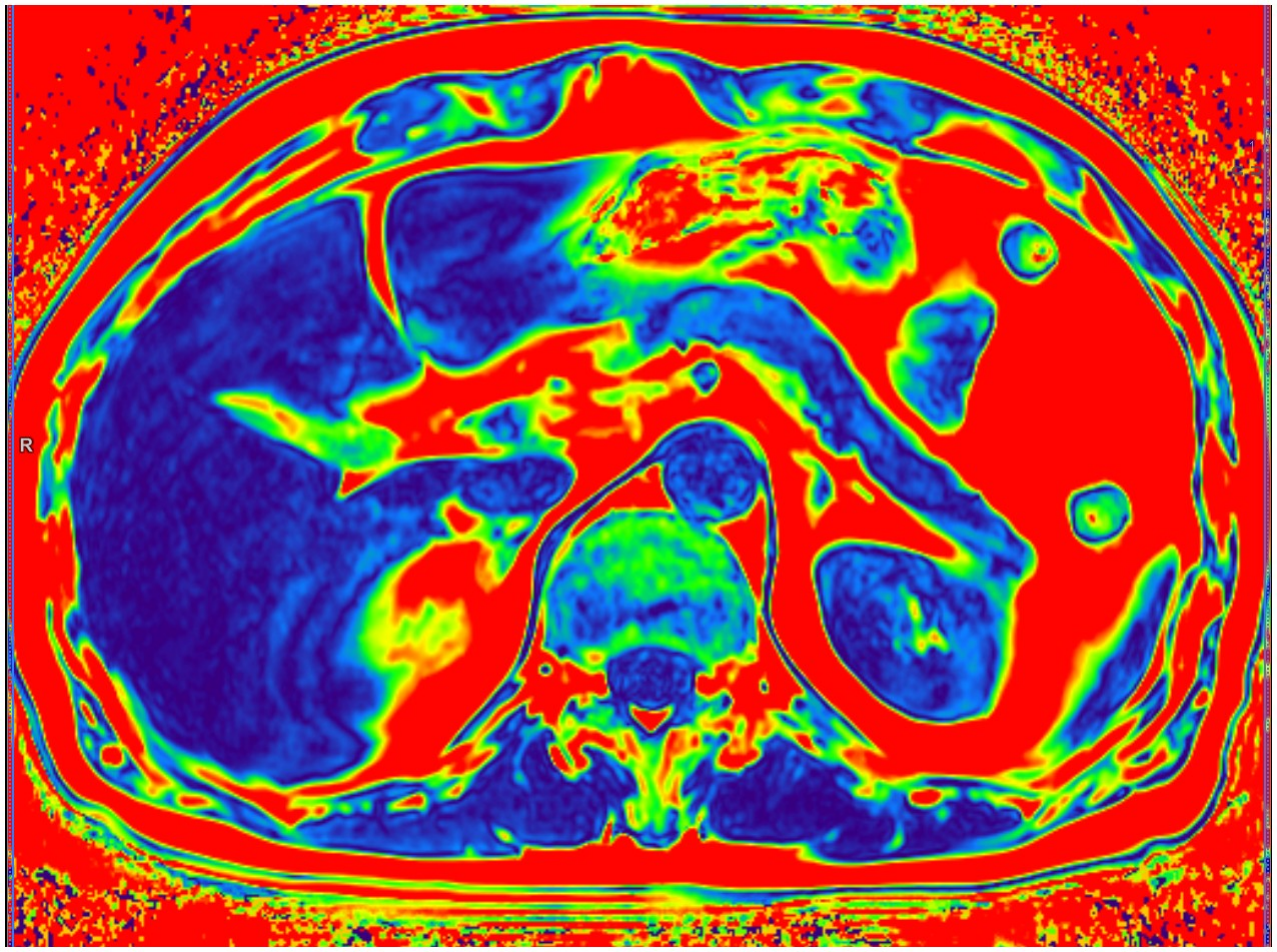
Color-coded T2 fat signal fraction map depicts areas of fat accumulation on the example of a healthy patient with BMI 21 where the dark blue color corresponds to the lowest fat content and the red to the highest fat content (calculated mean fat fraction: Liver– 2%, pancreas– 4%, muscles– 3%).

**Fig 6:**
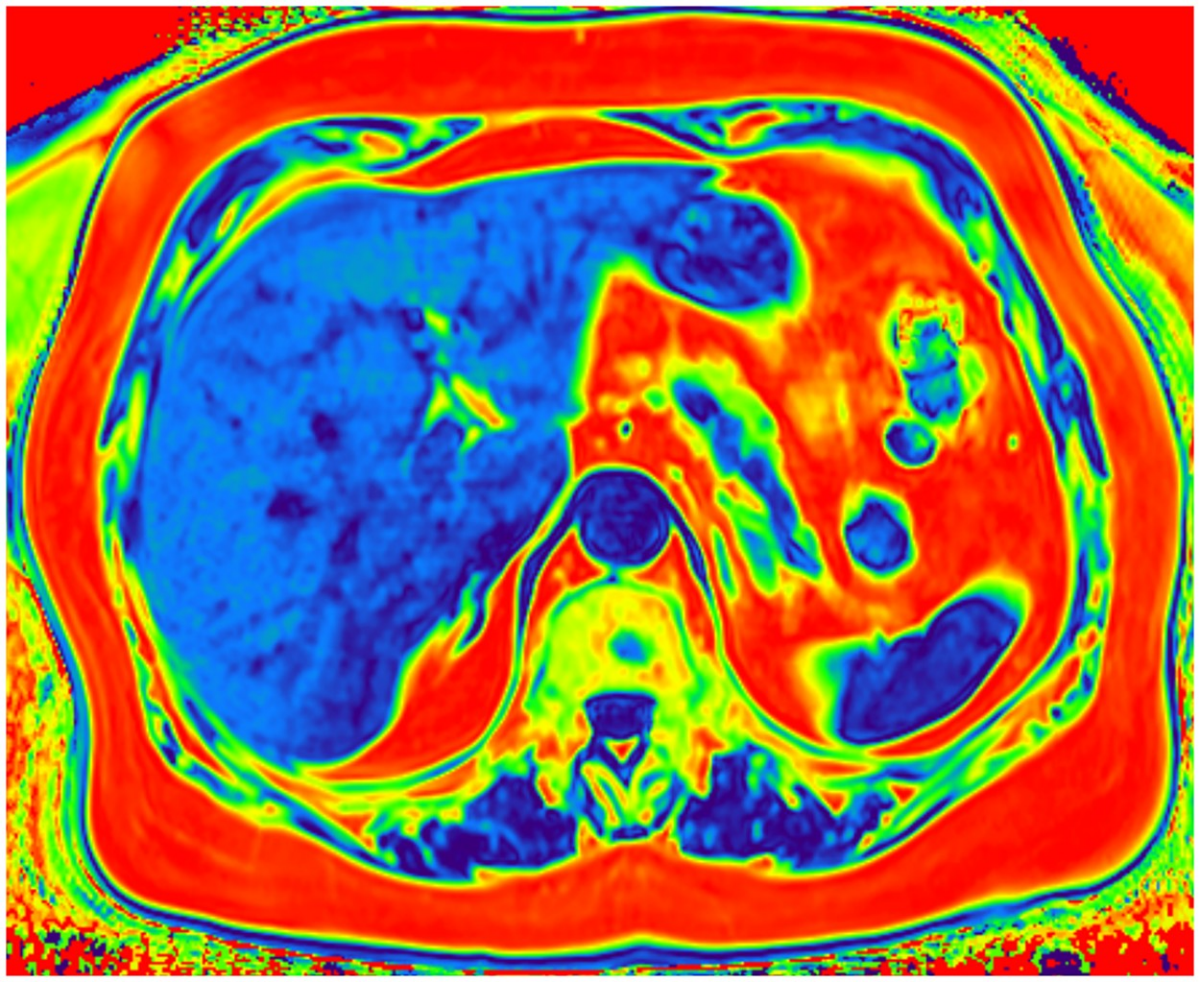
Color-coded T2 fat signal fraction map depicts areas of fat accumulation on the example of a patient with BMI 29 who was diagnosed with diabetes and hypertension where the dark blue color corresponds to the lowest fat content and the red to the highest fat content (calculated mean fat fraction: Liver– 21%, pancreas– 13%, muscles– 11%).

Two additional round ROIs were drawn on the bilateral paraspinal muscles at the lumbar vertebra 3 level, that is the level which is considered the best for both skeletal and visceral fat assessment in healthy middle-aged adults, as well as subcutaneous adipose tissue [20]. The average fat fraction for muscles is the arithmetic mean of the fat fraction in the right and left paraspinal muscle (Figs 1–6).

All measurements were carried out by two independent observers.

The general exclusion criteria were as follows: the use of steatogenic medications, significant systemic illnesses, age younger than 18 years, alcohol intake of more than 10g per day in the previous one year and the lack of consent to participate in the study.

### Statistical analysis

To assess the interdependence between the analyzed variables, Pearson’s correlation coefficients were calculated and regression lines presented with appropriate scatter plots. Differences between mean values in independent groups were examined using parametric Welch’s t-test or ANOVA. Additionally, in case of more than two groups, results of HSD post-hoc test were introduced. Normality assumption of data set was checked by Shapiro-Wilk test. The level of significance was set at α = 0.05. All statistical analyses were performed with Statistica version 13.1 (Dell Inc. 2016, data analysis software system).

## Results

The study includes 267 consecutive patients (119 men aged from 18 to 83, mean 52, and 148 women aged from 19 to 82, mean 55).

Using the ICC test—the intraclass correlation coefficient, a high agreement between observers was found—the intraclass correlation was 0.987.

The mean BMI in the group of men was 26 kg/m^2^ (range from 15.92 to 40.40) and similarly 26 kg/m^2^ (range from 17.42 to 38.16) in women. Of all subjects, 133 patients had normal BMI, 79 were overweight, and 55 were obese.

It was found that the average steatosis of pancreas, muscles and liver in patients with normal BMI was 5.98%, 5.25% and 3.66% respectively, in overweight patients 9.36%, 7.56% and 5.88%, and in obese patients 11.69%, 10.93% and 8.26% (Table 1).

**Table 1:** Characteristics of the subjects included in the study divided on the basis of BMI (normal weight—BMI 18.5–24.9, overweight—BMI 25.0–29.9 and obese—BMI ≥ 30) in terms of fat accumulation value in pancreas, muscle and liver.

Variable	ANOVA results						
	BMI group	N	Mean	Median	SD	F	p
**Pancreas fat fraction**	18.5–24.9	133	5.98	5,00	3,99	30.035	<0.001
	25.0–29.9	79	9.36	8,00	5,66		
	≥ 30	55	11.69	10,00	5,58		
**Muscles fat fraction**	18.5–24.9	133	5.25	4.00	4.14	20.275	<0.001
	25.0–29.9	79	7.56	7.00	5.31		
	≥ 30	55	10.93	8.00	8.46		
**Liver fat fraction**	18.5–24.9	133	3.66	2.83	2.67	18.244	<0.001
	25.0–29.9	79	5.88	4.10	6.19		
	≥ 30	55	8.26	5.88	6.62		

BMI—Body Mass Index.

Patients with normal BMI had an average waist circumference smaller than the reference values by 2.48 cm, overweight patients greater than the reference values by 10.7 cm, and in obese patients, the waist circumference exceeded the reference values by an average of 24.33 cm (Table 2).

**Table 2:** Deviation of waist circumference from reference value (CD, circumference deviation) in obese, overweight and normal BMI patients.

Variable	Descriptive statistics for CD					
	BMI group	N	Mean	Min	Max	SD
CD (cm)	Normal	133	-2.48	-24.00	22.00	9.91
	overweight	79	10.70	-9.00	33.00	9.46
	obese	55	24.33	8.00	51.00	9.29

CD—circumference deviation; BMD—Body Mass Index.

Using a variable determining the patient's waist circumference deviation from the reference value (CD), a statistically significant, moderately positive correlation between the degree of steatosis of particular organs, and expressed in centimeters difference between the waist circumference of the patient and the reference value was found (Table 3).

**Table 3:** Correlations between the degree of steatosis of particular organs and the value of variable determining patient's waist circumference deviation from the reference value (CD).

Variable	Pearson’s linear correlation coefficients (p-value)		
	Steatosis of pancreas [%]	Steatosis of muscles [%]	Steatosis of liver [%]
CD (cm)	0.429 (p<0.001)	0.500 (p<0.001)	0.405 (p<0.001)

CD—circumference deviation.

Analyzing the obtained values, the best correlation between the increase in the waist circumference in centimeters and the fat accumulation in muscles was demonstrated. It was revealed that increasing the waist circumference by 1 cm in relation to the reference values leads to an increase in steatosis of muscles by 0.211%. It has also been shown that when the waist circumference increases by 1 cm, the steatosis of the pancreas increases by 0.162% and the liver by 0.147%.

Of all patients, 93 had type 2 diabetes, and 91 had hypertension. In 56 people both diabetes and hypertension were diagnosed.

The average fat accumulation in pancreas, skeletal muscles and liver was estimated at 10.49%, 9.80% and 6.64% in diabetic patients, whereas in non-diabetic patients the fat content was significantly lower and amounted to 6.93%, 5.64% and 4.54%, respectively.

In hypertensive patients, the mean fat accumulation in pancreas was 10.76%, while 9.82% in muscles and 6.37% in liver.

The mean steatosis of pancreas, skeletal muscles and liver in patients without hypertension was significantly lower and amounted to 6.98%, 5.83% and 4.77%, respectively.

**Fig 7:**
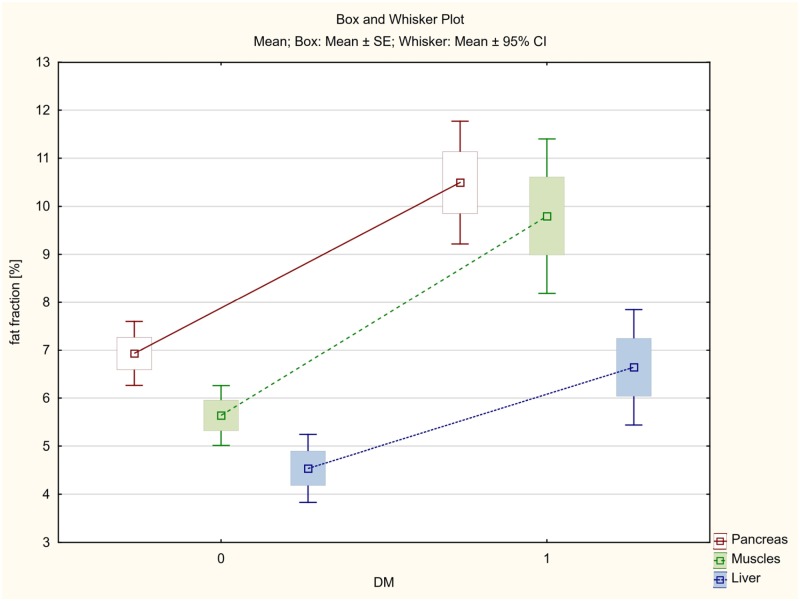
The average fat accumulation in pancreas, skeletal muscles and liver in the group of patients with type 2 diabetes (DM).

**Fig 8:**
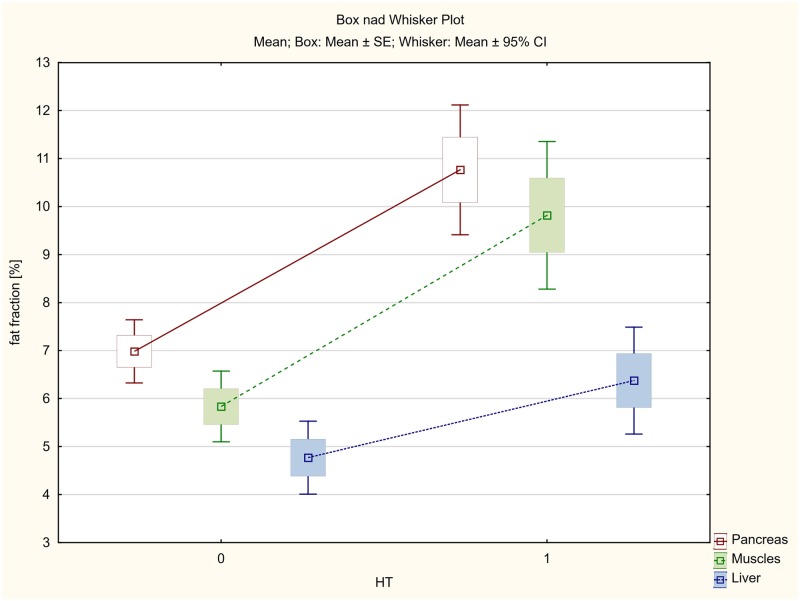
The average fat accumulation in pancreas, skeletal muscles and liver in the group of patients with hypertension (HT).

The smallest, although statistically significant, differences were found in the liver (Table 4, Figs 7 and 8).

**Table 4:** Fat accumulation in pancreas, skeletal muscles and liver in the group of patients with diabetes or hypertension in comparison to the non-diabetic and non-hypertension group.

Variable	Welch's t-test for independent groups T2DM = 1, without T2DM = 0, HT = 1, without HT = 0					
	T2DM = 0	T2DM = 1	P	HT = 0	HT = 1	P
**Pancreas fat fraction %**	6.931	10.495	<0.001	6.983	10.764	<0.001
**Muscle fat fraction %**	5.637	9.796	<0.001	5.834	9.818	<0.001
**Liver fat fraction%**	4.540	6.643	0.003	4.769	6.374	<0.019

T2DM—type 2 diabetes mellitus; HT—hypertension.

The differences between steatosis of pancreas, skeletal muscles and liver between three groups of patients: (1) patients with both diabetes and hypertension (DM & HT group), (2) patients with either diabetes or hypertension (DM or HT group) and (3) patients with no diabetes and no hypertension (None group), were also assessed.

Among patients with both diabetes and hypertension (DM & HT group), the average steatosis of pancreas, skeletal muscles and liver was respectively 11.39%, 10.61% and 6.70% and in the case of patients with type 2 diabetes only or hypertension only (DM or HT group), the fat accumulation was 7.32%, 6.16% and 4.90% respectively.

Even greater statistically significant differences in ectopic fat accumulations in the assessed organs were demonstrated between the group of patients with both diabetes and hypertension (DM & HT), and patients with no diabetes and no hypertension (None group), whose fat accumulation was 6.43%, 5.13% and 4.31% respectively.

**Fig 9:**
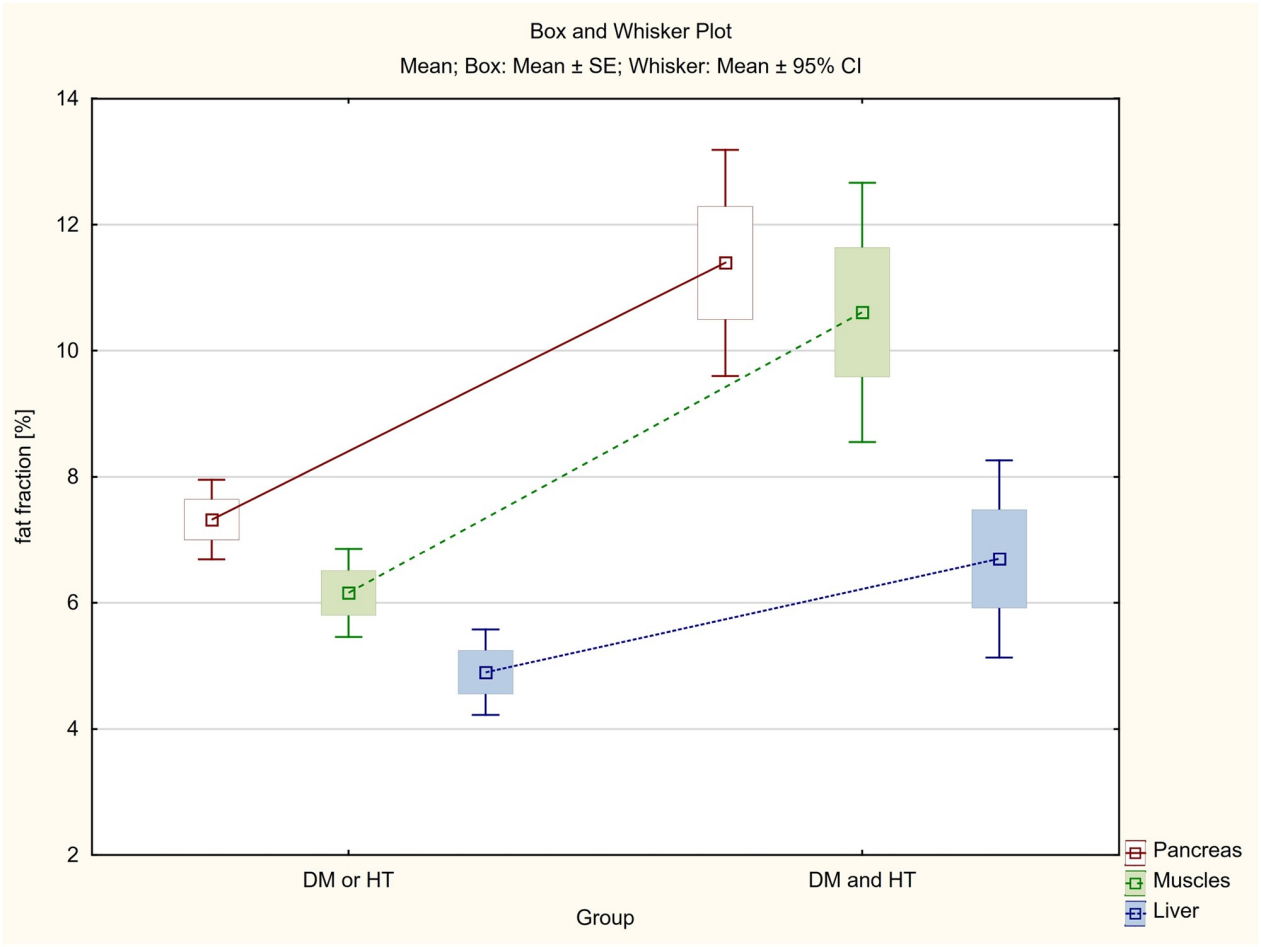
The differences between average fat accumulation in pancreas, skeletal muscles and liver between the group of patients with both diabetes and hypertension (DM and HT) and patients with either diabetes or hypertension (DM or HT group).

**Fig 10:**
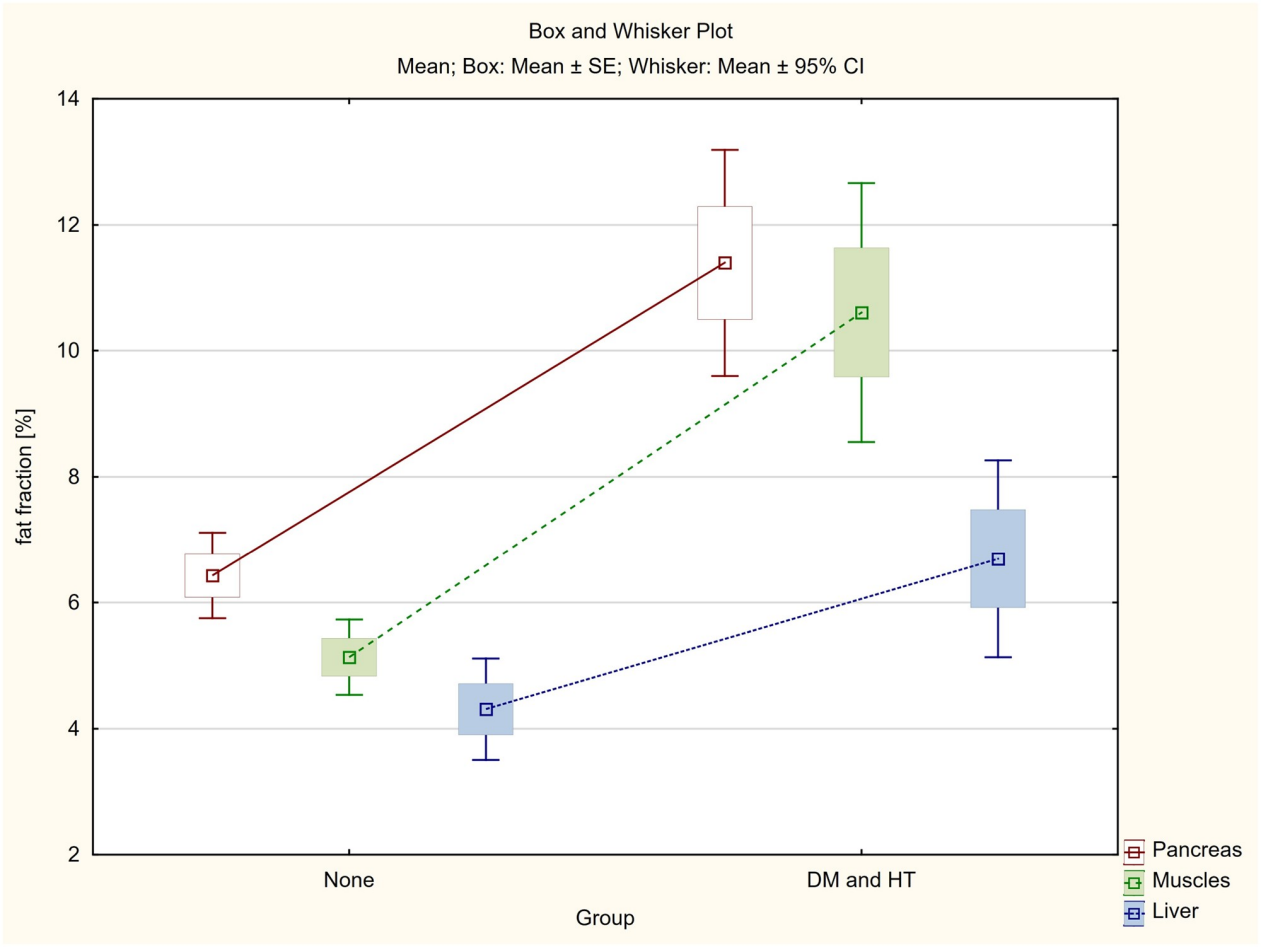
The differences between average fat accumulation in pancreas, skeletal muscles and liver between the group of patients with both diabetes and hypertension (DM and HT) and patients with no diabetes and no hypertension (None group).

As previously, the smallest, although statistically significant, differences were found in the liver (Table 5, Figs 9 and 10).

**Table 5:** Fat accumulation in pancreas, skeletal muscles and liver in the group of patients with both diabetes and hypertension (DM and HT) in comparison to the patients with either diabetes or hypertension (DM or HT group) and patients with no diabetes and no hypertension (None group).

Variable	Welch's t-test for independent groups					
	DM&HT	DM or HT	P	DM&HT	None	p
**Pancreas fat fraction %**	11.393	7.321	<0.001	11.393	6.430	<0.001
**Muscle fat fraction %**	10.607	6.156	<0.001	10.607	5.132	<0.001
**Liver fat fraction%**	6.697	4.898	0.038	6.697	4.309	0.008

Comparison of these values with the average steatosis in three BMI groups indicates that all values correspond to the BMI groups of people who are overweight or obese. BMI of 25 or above was calculated to be present in 73% of diabetic patients (68 out of 93 patients with diabetes, 32 of them being overweight and 36 obese), 74% of patients with hypertension (67 out of 91, 29 –overweight, 38 obese), and 75% of patient who suffered both from diabetes and hypertension (42 out of 56, 19 overweight and 23 obese).

Comparing the BMI and WCR values, statistically significant differences between the group of patients with diabetes (T2DM = 1) or with hypertension (HT = 1) and the group of patients without diabetes (T2DM = 0) or without hypertension (HT = 0) were found.

In patients with diabetes, the mean BMI was 28.02 kg/m^2^ and WCR 116.63%, while in non-diabetic group the mean BMI values was 24.86 kg/m^2^ and WCR 104.39%.

In patients with hypertension, the mean BMI was 27.65 kg/m^2^ and WCR 116.29% while in non-hypertensive patients these values were significantly lower and reached 25.19 kg/m^2^ and 105.16% respectively (Table 6).

**Table 6:** Mean value of BMI and WCR in the group of patients with diabetes or hypertension in comparison to the non-diabetic and non-hypertension group.

Variable	Welch's t-test for independent groups T2DM = 1, without T2DM = 0, HT = 1, without HT = 0					
	T2DM = 0	T2DM = 1	P	HT = 0	HT = 1	p
**BMI**	24.860	28.023	<0.001	25.187	27.655	<0.001
WCR	104.387	116.631	<0.001	105.156	116.287	<0.001

BMI—Body Mass Index; WCR—waist circumference ratio; T2DM—type 2 diabetes mellitus; HT—hypertension.

Both in diabetes and hypertension, it was shown that the waist circumference was on average higher than the reference value by over 16%.

## Discussion

According to WHO data, 3.2 million people worldwide die of complications related to diabetes each year, mainly due to the increased risk of cardiovascular diseases accounting for 80 per cent of those deaths [7, 17].

For years, MRI has been considered the most reliable and accurate method of non-invasive assessment of fat accumulation in the liver. Various MR techniques are used, such as magnetic resonance spectroscopy, in-phase and out-of-phase imaging, and finally a method of separating water and fat signals, based on chemical shift, used in our work and proposed by Dixon in 1984 (fat-water separated Dixon imaging technique). In the present study, we have assessed ectopic fat accumulation in pancreas, skeletal muscles and liver in 267 consecutive patients with obesity, overweight and normal BMI, in correlation with the presence of type 2 diabetes, hypertension and abdominal obesity.

It was estimated that the average steatosis of the pancreas, muscles and liver in patients with normal BMI was 5.98%, 5.25% and 3.66% respectively (Table 1).

The obtained values are consistent with other works assessing pancreatic fat content by magnetic resonance imaging.

For example, Heber [4] determined the average fat content in pancreas at 5.2%, Kuhn [21] at 4.4%, and Singh [17] considers that a cutoff point of 6.2% may be recommended for further studies as the threshold for normal fat content in the pancreas. Also, the hepatic fat accumulation value obtained by us in patients with normal BMI does not exceed the widely accepted hepatic fat threshold determined on the basis of liver biopsy and imaging examinations at 5% [17, 22, 23]. That value is the recommended fat threshold which makes it possible to diagnose non-alcoholic fatty liver disease (NAFLD).

In the available literature, we did not find any information on the cut-off points of fat accumulation in skeletal muscles.

Analyzing the obtained values of fat infiltration of pancreas, muscles and liver in people with overweight and obesity, a significant increase in fat accumulation was observed along with an increase in BMI. The organ most susceptible to fat deposition with the increase in BMI was the pancreas. It correlates with a study conducted on mice, which after 3–15 weeks of being on a high-fat diet, increased fat content in the pancreas but not in the liver [3, 24]. Interestingly, our study showed that muscles are also more susceptible to fat accumulation than the liver.

Considering the importance of the waist circumference, which has recently been observed as more indicative of the metabolic syndrome profile than BMI, and the validity of abdominal obesity as a risk factor for severe disease processes including type 2 diabetes [4, 25–27] we decided to introduce gender-specific variables. The first one, defining the patient's waist circumference deviation from the reference value expressed in centimeters and the second, shown in per cent and described as WCR [19]. It was calculated that in the obese group, the waist circumference was on average 24 cm higher compared to the norm, while in the overweight group it was almost 11 cm larger than the reference values (Table 2). Regarding the reference values, 1 cm increase of the waist circumference resulted in the enlargement of muscles steatosis by 0.211%, pancreas by 0.162% and liver by 0.147%. It confirms the previously obtained result that the liver is less susceptible to steatosis compared with the pancreas and skeletal muscles (Table 3).

In the present study we obtained statistically significant differences in mean steatosis of all the assessed organs in the group of patients with diabetes or hypertension in comparison with the group without diabetes and hypertension (Table 4, Figs 7 and 8).

Both in the case of T2DM and HT, the differences were more considerable for the pancreas and muscles (p<0.001), and once again smaller, although also statistically significant, for the liver (p<0.019).

Dividing all patients into three groups, the first of which were patients with both diabetes and hypertension (DM & HT group), the second patients with only diabetes or only hypertension (DM or HT group) and the third one with non-diabetic and non-hypertensive subjects (None group), statistically significant differences were found between fat infiltration of the assessed organs (Table 5, Figs 9 and 10). In the case of patients diagnosed with both diabetes and hypertension (DM & HT group), the average steatosis of the pancreas, skeletal muscles and liver was significantly higher not only than in the group without diabetes and hypertension (None group), but also in patients diagnosed only with diabetes or hypertension (DM or HT group). Again, the differences were highest for the pancreas and muscles (p<0.001), and distinctly smaller although statistically significant for the liver (p<0.008 and p<0.038 respectively).

Analyzing BMI and WCR values in patients with diabetes and hypertension, significantly higher BMI values were observed in both cases compared to the healthy group. A similar association occurred in the case of WCR, which in both diabetes and hypertension was over 16% higher concerning the reference values (Table 6).

Despite the lack of work showing cut-off points for clinical symptoms in the case of pancreatic steatosis, the results clearly show that in patients with diabetes and hypertension, the fat accumulation in individual organs is statistically more significant.

The obtained results are in line with the work of Pitt, Lingvay, Wang and Yu [3, 28–30] who also found higher fat content in the pancreas in people with T2DM compared with non-diabetic subjects. In the work of Wu [31], including 557 people, it was demonstrated that people with steatosis of the pancreas are more likely to develop hypertension, dyslipidemia and hyperglycemia in comparison with subjects without ectopic fat accumulation. According to Yamazaki [16] in the study involving up to 813 people, it was shown that fat content in pancreas is positively associated with T2DM in univariate analysis but with potentially confounding factors such as age, sex, BMI or alcohol abuse. Also, the number of met MetS criteria, which include both diabetes and hypertension, increases with the degree of fatty infiltration in the individual organs, which is mentioned, inter alia, in the works of Lee, Sepe and Lesmana [15, 32, 33].

In contrast to the paper of Tushuizen [34] in our study, similarly to other studies [3, 14, 32, 35, 36], fat content in pancreas correlates with both BMI and waist circumference.

In the literature we can find information about the lack of a confirmed connection between pancreatic fat infiltration and beta cell function regardless of the glucose tolerance status, and the pancreas steatosis is not independently associated with future appearance of T2DM [3, 14, 16, 37]. Also, Kühn [21] did not prove a relationship between the content of fat accumulation in the pancreas and impaired glucose metabolism.

On the other hand, it is confirmed that people with steatosis of the pancreas are characterized by insulin resistance and that is believed to be a good predictor of T2DM and may precede the clinical onset of diabetes for many years [3, 6, 14].

It seems, therefore, that non-invasive MRI examination allowing the assessment of lipid content in individual organs may become an ideal tool to help struggle with obesity consequences.

Of course, further researches are required to show what the clinical implications of fat accumulation in particular organs are, both regarding their damage and systemic effects, and an attempt should be made to determine the cut-off points for clinical symptoms in the case of fatty infiltration of individual organs.

## Conclusions

In conclusion, we have revealed that there is a relation between the fat content in muscles, pancreas and liver, the incidence of type 2 diabetes and hypertension and also body mass index and waist circumference ratio.

It is uncertain whether the use of fat content measurement in some organs could also be used as a biomarker, which would be able to indicate the insulin resistance that develops during obesity, or whether MRI can enable early detection of reversible metabolic changes, as well as their subsequent monitoring.

It seems that future studies should aim at establishing whether the measurement of fat content in some organs can also be used for the abovementioned purposes.

## Supporting information

S1 Data (XLSX)

## List of abbreviations

BMI: body mass index
CD: circumference deviation
FF: fat fraction
HDL: high-density lipoprotein
HT: hypertension
LDL: low density lipoprotein
MetS: metabolic syndrome
MRI: magnetic resonance imaging
NAFLD: non-alcoholic fatty liver disease
ROI: region of interest
SI: signal intensity
T2DM: type 2 diabetes mellitus
TG: triglyceride
WCR: waist circumference ratio
WHO: World Health Organization
